# Impact Angle–Velocity Interactions Governing Solid-Particle Erosion in Laser Powder Bed Fused AlSi10Mg

**DOI:** 10.1021/acsomega.5c13185

**Published:** 2026-07-30

**Authors:** Rashmi Saragur Nanjundaiah, Shrikantha Sasihithlu Rao, Praveenkumar K, Calvin Samuel S, T. Ram Prabhu, Katerina Skotnicova

**Affiliations:** † Department of Mechanical Engineering, 119680 National Institute of Technology Karnataka, Surathkal 575 025, Karnataka, India; ‡ Department of Industrial and Production Engineering, The National Institute of Engineering, Mysore 570 008, Karnataka, India; § Faculty of Materials Science and Technology, VSB-Technical University of Ostrava, 17. listopadu 2172/15, 70 800 Ostrava, Czech Republic; ∥ Department of Design and Manufacturing, 29120 Indian Institute of Science, Bengaluru 560012, Karnataka, India; ⊥ Centre for Military Airworthiness and Certification, Defence Research and Development Organization, Bengaluru 560 037, Karnataka, India

## Abstract

This study investigates the erosive wear behavior of laser powder bed fused (LPBF) AlSi10Mg alloy samples subjected to different heat treatments, including T5 (direct aging), T6, SR (stress relieving), and as-built (AB) conditions. The erosion study was carried out under different impact angles (30, 60, and 90°) and different impact velocities (30, 70, and 90 m/s). The erosive wear is severe at 30° impact angle, indicating that the ductile mode is prominent. The wear rate increases for all materials as the impact velocity increases from 30 to 90 m/s. The T5 heat-treated sample consistently exhibits the lowest erosive wear rate. In contrast, the T6 heat-treated and as-built (AB) samples display higher susceptibility to erosion, influenced by their properties. SEM analysis highlights distinct surface features across the samples; T5 samples show deep grooves and material smearing, indicating effective absorption of impact energy at 90° impact angle, while T6 and AB samples exhibit evidence of brittle fracturing, such as fragmented particles and debris. The critical findings from the study highlight that microstructural stability is also a key factor in erosion behavior alongside hardness, emphasizing the importance of heat treatment in optimizing wear resistance for high-impact applications.

## Graphical abstract

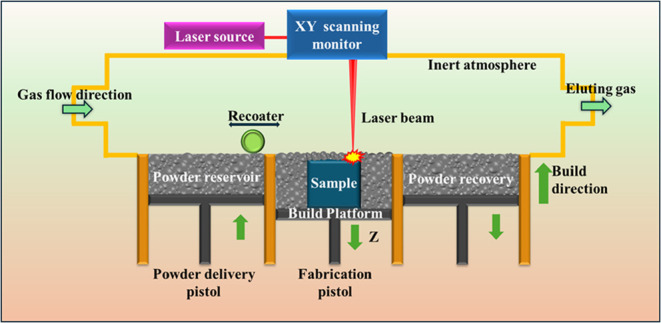

## 1 Introduction

AlSi10Mg is used in the aerospace and automotive industries for producing components such as inlet manifolds, brackets, housings, and heat sinks.^1,2^ The main characteristics of the alloy are low density, good mechanical strength, high thermal conductivity, and corrosion resistance, which make it a highly dynamic resilience alloy that contributes to improved performance and efficiency. AlSi10Mg has a significant amount of Si and Mg content that gives high weldability and makes it suitable for an additive manufacturing (AM) process.^3^ The AM process constructs parts by sequentially adding contoured layers, based on the geometry of the digital 3D CAD model.^4–6^ The AM process is widely adopted by the industries due to its numerous advantages such as material efficiency, lightweight manufacturing through shape optimization and lattice structures, near net shape, and design freedom.^6^ The combination of the AlSi10Mg alloy’s lightweight nature and versatility, alongside AM processes, makes it a top choice for aerospace, medical, and high-performance industries.^6^ The design freedom in the AM process enables the creation of intricate geometries, resulting in part integration that minimizes assembly time and, thereby, the lead time. Also, the intricate manufacturing capability in the AM process results in the production of lightweight components through shape optimization and generative design techniques, making it a sustainable manufacturing method by minimizing resources such as material and energy.^7^

Among various AM techniques, the laser powder bed fusion (L-PBF) process is a highly capable technique compared to other AM techniques due to its numerous advantages. It enables the fabrication of intricate geometries with high precision and resolution, surpassing methods like DED or binder jetting.^1,5,8–10^ Due to the rapid solidification, the L-PBF process produces parts with a fine microstructure, resulting in high strength and enhanced fatigue resistance. Additionally, L-PBF delivers a superior surface finish, reducing postprocessing requirements, and its high material utilization minimizes waste as excess powder can be reused. The L-PBF process offers broad material compatibility and high resolution, attributed to its small laser spot size (0.1 mm) and layer height (20–100 μm).^11,12^ In this process, a high-power focused laser source melts and fuses metal powder particles to create layers that are subsequently built up to form a part. Moreover, AlSi10Mg parts produced via L-PBF exhibit excellent thermal stability and corrosion resistance, making them suitable for use in heat exchangers and aerospace components. Overall, L-PBF is preferred for manufacturing high-performance, lightweight, and complex AlSi10Mg components with outstanding mechanical and thermal properties.

The successful application of L-PBF for processing aluminum alloys has primarily been achieved with Al–Si alloys. In Al–Si alloys, the addition of silicon lowers the melting temperature and reduces shrinkage, while the presence of magnesium makes these alloys heat-treatable, making them suitable for structural applications.^13^ In the L-PBF process, exceptionally fine microstructures are common due to extremely high cooling rate and temperature gradient. In Al–Si alloys, a silicon network surrounding Al cells is typical. High cooling rates lead to a high degree of silicon supersaturation in Al cells. Fine silicon particles, Mg_2_Si precipitates, and entangled dislocations are present in the aluminum matrix, contributing to material strengthening through various mechanisms, resulting in high mechanical properties, particularly strength and hardness. Heat treatment significantly influences the alloy’s microstructure, tailoring properties such as strength and ductility for various applications through controlled precipitation and phase dissolution.^14^ Heat treatment is essential for many high-performance alloys, particularly Al–Si alloys produced by L-PBF, as they tend to have high residual stresses and anisotropy.^15^ This postprocessing step is necessary to improve their properties, which can enhance strength, ductility, and overall performance.^16^ Heat treatment allows tailoring the alloy for different applications by adjusting its properties through controlled precipitation and dissolution of phases. Extensive research studies were carried out to investigate the impact of heat-treatment parameters on the microstructural and mechanical properties of Al–Si alloys. Previous studies on LPBF AlSi10Mg have employed different heat-treatment schedules depending on the targeted microstructural response and mechanical performance. Typical T5 aging treatments are generally conducted within 160–180 °C for several hours, whereas T6 treatments commonly involve solution treatment in the range of 500–540 °C followed by artificial aging. Similarly, stress relief treatments are often performed between 250 and 350 °C to reduce residual stresses while preserving the microstructure. In the present study, the specific heat-treatment parameters were selected based on preliminary optimization and literature guidance to achieve stable microstructural conditions suitable for erosion investigations.^17,18^

In aerospace and automotive applications, the AlSi10Mg components are exposed to harsh environments, and the solid-particle erosion study helps to predict the alloy’s performance under real-world conditions.^19^ Solid-particle erosion is a form of wear that occurs when the solid particle makes contact/impact with solid surface^6,20^ The repetitive impact of solid particles causes surface damage, leading to the removal of material from the surface. The severity of erosion depends on several factors such as particle velocity, angle of impact, particle properties (size, shape, hardness), and the material of the surface being eroded.^20,21^ Many research studies reveal that the hard material shows resistance to abrasive wear damage more than the soft material.

While previous studies on L-PBF AlSi10Mg have investigated erosion behavior, they often consider either impact angle or particle velocity independently and rarely examine their combined influence across different postprocessed conditions. This study addresses this gap by systematically investigating the coupled effect of impact angle and particle velocity on the erosion response of L-PBF AlSi10Mg under multiple postprocessing conditions (as-built, T6, and stress-relieved). Experiments were conducted at velocities of 30, 70, and 90 m/s and at impact angles of 30, 60, and 90°, while maintaining a reference velocity of 70 m/s for comparison. Subsequent analyses, including quantitative mass-loss measurements and scanning electron microscopy, were performed to elucidate the dominant erosion mechanisms and correlate them with the imposed test conditions. This approach provides a comprehensive understanding of how postprocessing and impact parameters interact to influence erosion resistance, establishing predictive trends for the design of wear-resistant AM components.

## 2 Experimental Procedure

### 2.1 Material Preparation and Heat Treatment

The samples were built with LPBF using an EOSINT M 280 3D Printer equipped with a 400 W ytterbium fiber laser. Figure 1 represents the schematic diagram of the LPBF process system. Samples were built to a cubical shape with 15 × 15 × 8 mm^3^ dimensions. The study selected specific optimum parameters based on pilot experiments and earlier investigations.^22,23^ Before printing, the platform was heated to 30 °C. The gas-atomized AlSi10Mg powders used had spherical morphology with an average particle size of 15–30 μm. AlSi10Mg was manufactured in an inert atmosphere by purging with 99% argon gas, minimizing the effect of oxygen on its material properties.

**Figure 1:**
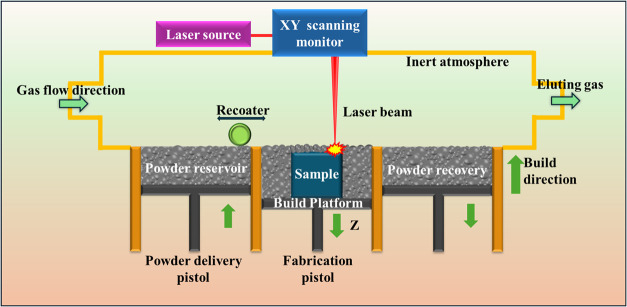
Schematic of the LPBF process.

Table 1 provides a summary of the optimized printing parameters. After printing, the test samples were machined out from the substrate using the wire electric discharge machining (WEDM) process. These samples were cleaned to remove residual powder or surface contaminants.

**Table 1:** LPBF Printing Parameters

parameters	set value
laser power (W)	370
scanning speed (mm/s)	1300
scanning strategy	rotating stripe (67°)
inert gas	Ar
built platform temp (°C)	30
layer thickness (μm)	70

Three different heat treatment processes, T5, T6, and Stress Relief (SR), were carried out to evaluate their effects on the erosion performance of the alloy. For the T5 treatment, the samples were placed in a furnace preheated to 170 °C and held at that temperature for 3 h. After the soaking period, the samples were allowed to cool slowly inside the furnace. The T6 treatment involved a two-step process: solution treatment followed by artificial aging. In this case, the samples were heated to 540 °C and held for 2 h, then immediately quenched in water at room temperature. After quenching, the samples underwent aging at 170 °C for 3 h, followed by furnace cooling. For the SR treatment, the samples were heated to 320 °C and held for 3 h, followed by furnace cooling to reduce internal stresses without significantly altering the microstructure. The selected heat-treatment conditions were adopted from preliminary optimization studies conducted on the same LPBF AlSi10Mg material system. Although slight variations from commonly reported industrial T5, T6, and SR schedules exist, the selected parameters were chosen to achieve the desired microstructural modifications and to maintain consistency with our previous investigations on the same processing route. After each heat treatment, the samples were subjected to microhardness and erosion study. The detailed methodology is shown in Figure 2.

**Figure 2:**
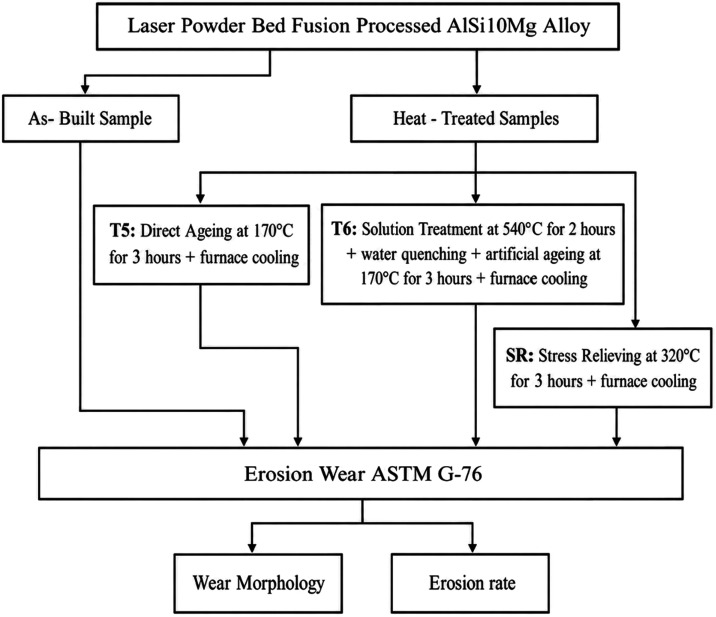
Comprehensive schematic representation of the different test processes.

### 2.2 Electron Backscatter Diffraction (EBSD)

The crystallographic characteristics of the samples were examined using EBSD to evaluate the grain structure, texture, misorientation distributions, kernel average misorientation (KAM), and grain size. Prior to EBSD analysis, specimens were prepared by sequential mechanical grinding using SiC papers from no. 220 to no. 2500 grit, followed by final polishing with diamond paste to obtain a mirror-like surface finish. The polished surfaces were subsequently subjected to electrolytic polishing using an A2 electrolyte composed of 100 mL of butoxyethanol, 730 mL of ethanol, 78 mL of perchloric acid, and 70 mL of distilled water. EBSD measurements were performed on cross sections using a Helios Nanolab dual-beam scanning electron microscope (FEI). The analyses were conducted at an accelerating voltage of 25 kV and a beam current of 15 nA. Post-acquisition data processing, including indexing and orientation map generation, was carried out using TSL-OIM 8 software.

### 2.3 Microhardness

Vickers microhardness tests of as-built samples and heat-treated samples were performed using an Omni Tech microhardness tester. The test was carried out at a load of 100 g with a dwell time of 10 s. The hardness was calculated based on the applied load and the projected area of the indentation produced by the diamond indenter. A minimum of 10 indentations were measured and averaged for the report; the standard deviation was considered as an error bar.

### 2.4 Erosion Study

Erosive wear tests were conducted on as-printed and heat-treated samples using a DUCOM Erosion wear testing machine under the guidelines of ASTM-G76 standards.^24^ The erosion study was carried out in two sets: (1) Varying angle (30, 60, and 90°) for constant velocity (70 m/s) and (2) Varying velocity (30, 70 and 90 m/s) for constant angle (30°). The erosion test conditions (30, 60, and 90°) were selected to capture different erosion mechanisms of the AlSi10Mg alloy. For ductile materials, the maximum erosion rate generally occurs at low impact angles (∼20–30°) because the tangential component of particle velocity promotes microcutting and plowing of the surface. In contrast, near-normal impact (∼90°) promotes deformation and fracture mechanisms that are more typical of brittle behavior. Therefore, testing at 30, 60, and 90° enables the investigation of low-angle cutting wear, intermediate mixed-mode erosion, and normal-impact deformation within a single experimental framework. In addition to the impact angle, particle velocity significantly influences erosion behavior. Higher particle velocities increase the kinetic energy transferred to the target surface, leading to enhanced material removal, severe plastic deformation, and possible subsurface crack formation. At very high velocities, particle rebound may also occur, which can influence the interaction of subsequent particles with the surface and thus affect the overall erosion rate. Therefore, three velocities (30, 70, and 90 m/s) were selected to systematically evaluate the velocity-dependent erosion characteristics of the LPBF AlSi10Mg alloy, which has not been extensively studied under these specific conditions. The velocity study was performed at 30° impact angles, which produced the highest erosion rate during the angle-dependent experiments. Consequently, the results primarily represent velocity effects under the most severe erosion condition rather than a complete angle–velocity interaction map.

The printed test samples with dimensions of 15 mm × 15 mm × 8 mm were subjected to erosion study. Alumina (Al_2_O_3_) particles of average size 50 μm were used as an erodent. Erosive wear rate was measured by the weight loss technique. Erodent feed rate was maintained at 2 g/min throughout the study. A 1.5 mm diameter nozzle was used in the erosion tester, and the stand-off distance was maintained at 10 mm. Each test was carried out for a duration of 10 min. Prior to the test, the test samples were cleaned with acetone to remove grease and oil contamination from the surface. The test samples were sequentially polished up to #2500 grit using Sic abrasive papers, followed by velvet cloth polishing assisted with diamond aerosol spray to obtain a mirror finish. The mirror-polished surface captures the erosion features created by the erodent, which helps in understanding the erosion mechanism under different testing conditions. After the erosion test, the samples were subjected to the scanning electron microscope FE-SEM (Quanta 250 FEG) to further understand the mechanism.

## 3 Result and Discussion

### 3.1 Overview of Our Previous Investigations on AlSi10Mg

Previous studies on LPBF-fabricated AlSi10Mg^2,25^ investigated microstructure, phase composition, residual stress, and wear behavior. Key observations relevant to the current erosion study are summarized as follows:

a. The as-built material exhibited a hierarchical microstructure with fine cellular α-Al structures and silicon particles, with distinct features at melt pool centers and boundaries.
b. Heat treatments such as T5 and T6 led to partial dissolution of the cellular α-Al structure and more uniform dispersion of Si particles, refining the microstructure.
c. SR treatment preserved the original Si particle distribution while retaining most of the as-built structural features.
d. Residual stress measurements showed tensile stresses in the as-built state (∼30 MPa), whereas compressive stresses were observed after heat treatments: −7 ± 1.5 MPa for T5, −15 ± 2.2 MPa for T6, and −12 ± 1.8 MPa for SR-treated specimens.

The residual stress values and associated microstructural characteristics discussed herein are derived from our previous investigations performed by using the same LPBF processing parameters and heat-treatment schedules. These results are used to support the interpretation of the erosion behavior; however, residual stress was not directly measured in the present study. Therefore, the present discussion primarily emphasizes those features relevant to erosion performance, while the complete experimental details and characterization results are provided in refs. 2,25,2,25

### 3.2 Microstructure

The optical micrographs of the AlSi10Mg alloy under different processing conditions (Figure 3) reveal clear microstructural evolution from the as-built to the heat-treated states along with the build direction. In the as-built condition, the microstructure exhibits a fine cellular-dendritic network formed due to the extremely high cooling rates during the printing process, where a continuous eutectic Si network surrounds the α-Al matrix along the melt pool boundaries. This rapid solidification results in a supersaturated solid solution of Mg and Si and produces a fine microstructure that provides high strength but limited ductility owing to the brittle and interconnected Si network.^26^ After T5 treatment, the overall cellular morphology remains visible, although the Si network appears slightly coarsened and less continuous, indicating partial spheroidization and precipitation of Mg_2_Si during artificial aging without prior solution treatment. In the SR condition, the microstructure shows reduced definition of the melt pool boundaries and a more fragmented Si network, suggesting diffusion-induced homogenization and relief of residual stresses accumulated during the additive process. Finally, the T6-treated sample exhibits a fully transformed and homogenized microstructure, characterized by a uniform dispersion of fine, spheroidized Si particles within the α-Al matrix, with the disappearance of melt pool boundaries. This evolution occurs as a result of Si and Mg dissolution during solution treatment, followed by controlled precipitation during artificial aging. The transition from a cellular-dendritic as-built structure to a more equiaxed and spheroidized morphology in the T6 state demonstrates the strong influence of thermal treatments on phase distribution and solidification features, allowing optimization of mechanical behavior through microstructural control.^27,28^ Further, the detailed microstructural evolution is discussed through EBSD analysis.

**Figure 3:**
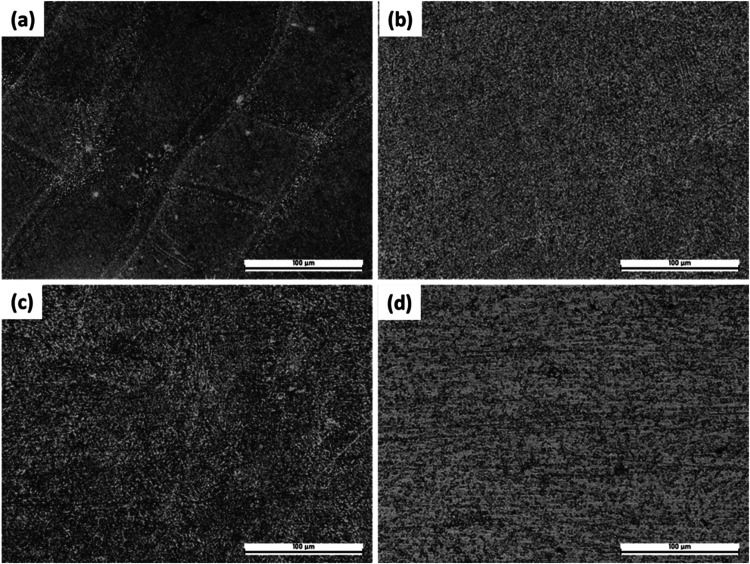
Optical micrographs of (a) as-built, (b) T5, (c) SR, and (d) T6 conditions.

### 3.3 Microstructural Evolution

The EBSD inverse pole figure (IPF) maps (Figure 4) reveal significant differences in grain morphology and crystallographic orientation among the four processing conditions, with different colors corresponding to specific crystal directions. The as-built microstructure (Figure 4a) is characterized by fine equiaxed grains with a strong degree of heterogeneity, typical of the rapid solidification and steep thermal gradients inherent to the LPBF process. The presence of irregular grain boundaries and cellular features indicates a high density of residual stresses, which is consistent with the as-built state. In contrast, the T5 condition (Figure 4b) shows partial grain coarsening and a reduction in the orientation scatter. The preservation of a relatively fine grain size suggests that the direct aging treatment stabilizes the microstructure without extensive recrystallization. This retained fine-grained morphology, combined with precipitation hardening, explains the improved wear resistance observed in the erosion testing. The SR condition (Figure 4c) exhibits elongated grains aligned along the build direction, reflecting partial recrystallization and stress relaxation. The grain boundaries appear to be more defined, and the reduction in residual stress likely promotes microstructural stability under mechanical loading. The elongated grain structure, however, introduces anisotropy that may influence the erosion response, particularly under oblique impact conditions. The T6 condition (Figure 4d) exhibits a comparatively homogenized microstructure with reduced cellular features and the presence of larger recrystallized regions relative to those of the as-built condition. Although the EBSD grain size distribution reveals a dominant population of fine grains, the overall microstructure shows reduced heterogeneity and diminished ultrafine cellular boundaries due to solution treatment and subsequent aging. Complete solutionizing followed by artificial aging reduces the fine cellular substructures, leading to a homogeneous microstructure. While this reduces internal stresses, the loss of ultrafine cellular boundaries influences the resistance to crack initiation during the erosion.

**Figure 4:**
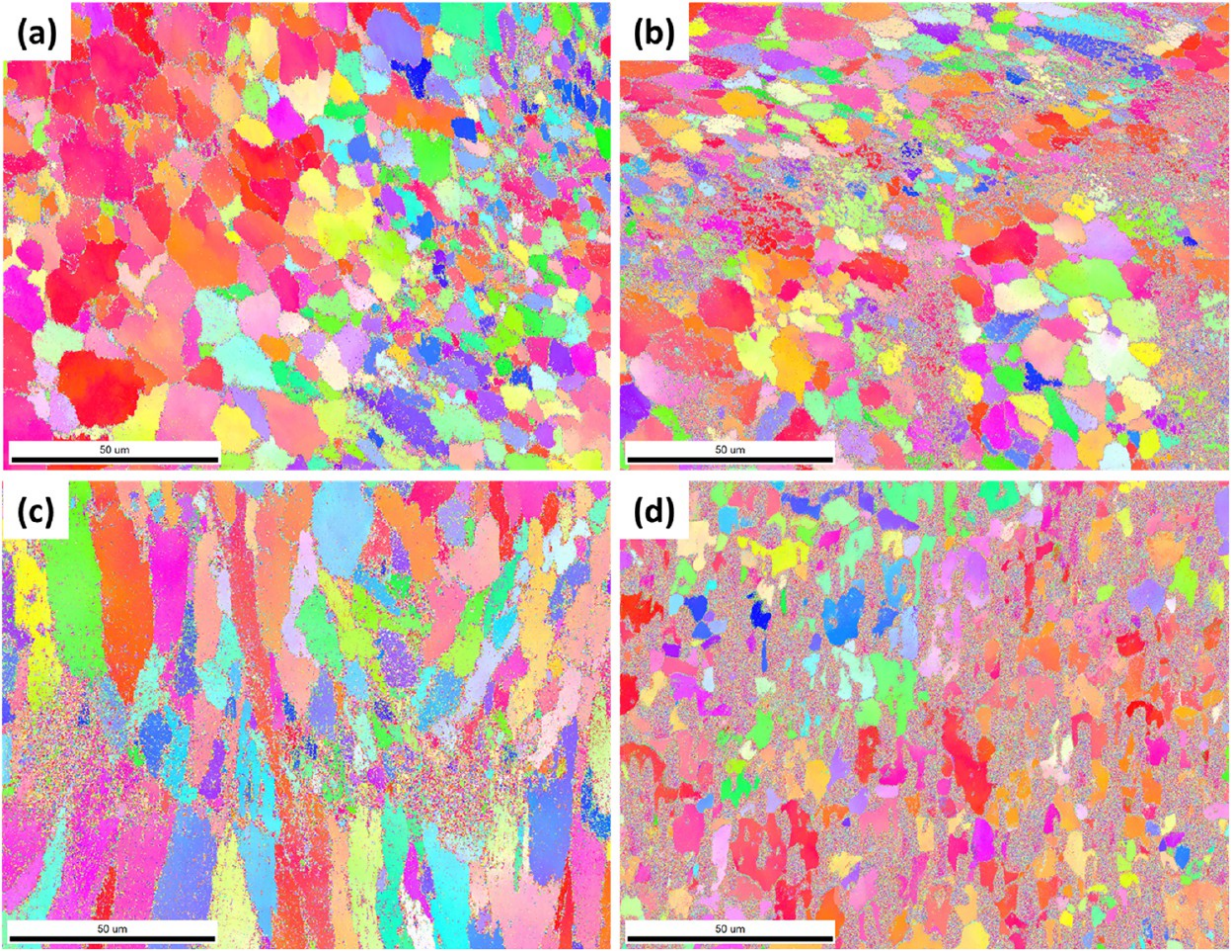
Inverse pole figure (IPF) orientation maps of LPBF AlSi10Mg in the (a) as-built, (b) T5, (c) SR, and (d) T6 conditions. The color coding represents crystallographic orientation according to the standard IPF color key shown in the figure.

The grain size distribution curves (Figure 5a) highlight the influence of postprocessing conditions on grain refinement and coarsening behavior. The as-built state shows a relatively broad grain size range, with a significant population of grains in the 5–15 μm regime. This broad distribution is consistent with the heterogeneous solidification conditions typical of LPBF, where thermal gradients vary across melt pools.^29^ After direct aging (T5), the distribution shifts toward finer sizes, with a notable presence of submicron grains. This refinement, along with the reduced spread in distribution, indicates that T5 stabilizes existing grains and promotes limited nucleation of fine precipitate-stabilized grains rather than extensive growth. In contrast, the SR condition displays a bimodal distribution with both submicron grains and coarser grains exceeding 10 μm. The presence of two distinct populations reflects partial recrystallization, where some grains grow preferentially while others remain fine, contributing to anisotropy in microstructural features. The T6 condition exhibits a narrower grain-size distribution compared with the as-built state, although EBSD maps reveal the presence of relatively coarser recrystallized grains. This apparent difference arises from the reduction of subgrain structures and homogenization of the microstructure following solution treatment and aging.

**Figure 5:**
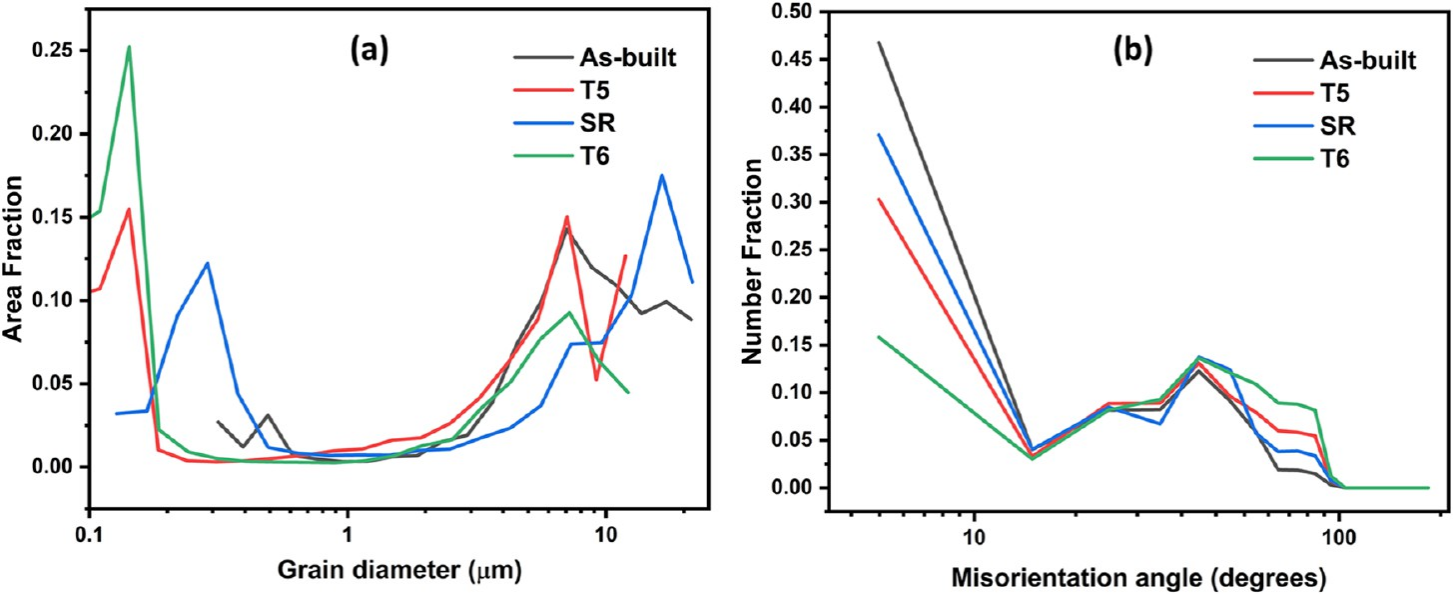
(a) Grain size distribution and (b) grain boundary misorientation angle fraction of LPBF AlSi10Mg in the as-built, T5, SR, and T6 conditions. The distributions illustrate the evolution of grain refinement and boundary characteristics following different heat treatments.

The misorientation angle fraction results (Figure 5b) provide further insight into the boundary characteristics across conditions. In the AB state, a high fraction of low-angle grain boundaries (LAGBs, < 10°) is evident, consistent with a high density of dislocations and subgrain structures produced by rapid solidification. The proportion of LAGBs decreases systematically with postprocessing, indicating progressive dislocation annihilation and stress relaxation. T5 and SR conditions maintain a balance between low- and high-angle grain boundaries (HAGBs), suggesting partial retention of substructural boundaries that may help resist crack propagation while reducing internal stress. In contrast, the T6 condition exhibits the lowest LAGB fraction, with a corresponding dominance of HAGBs, confirming extensive recrystallization and microstructural homogenization. While such boundary evolution effectively reduces stored strain, the reduction in LAGBs diminishes the potential barriers for dislocation motion, partially explaining the inferior erosion resistance of the T6 alloy compared to T5.

### 3.4 Local Misorientation and Stored Strain

Figure 6 presents the kernel average misorientation (KAM) maps, which represent the average local misorientation between a measurement point and its neighboring points. KAM is commonly used as a qualitative indicator of local lattice curvature and plastic strain, and higher KAM values correspond to regions with increased dislocation density and stored deformation. In the color scale, blue regions indicate low local misorientation (low strain), whereas green to red regions correspond to progressively higher misorientation angles, expressed in degrees. The AB sample exhibits elevated KAM values across most regions, confirming the high dislocation density induced by rapid solidification and thermal cycling during the LPBF. These localized strain fields are potential sites for crack initiation during erosion, explaining the brittle fracture features observed on worn surfaces.

**Figure 6:**
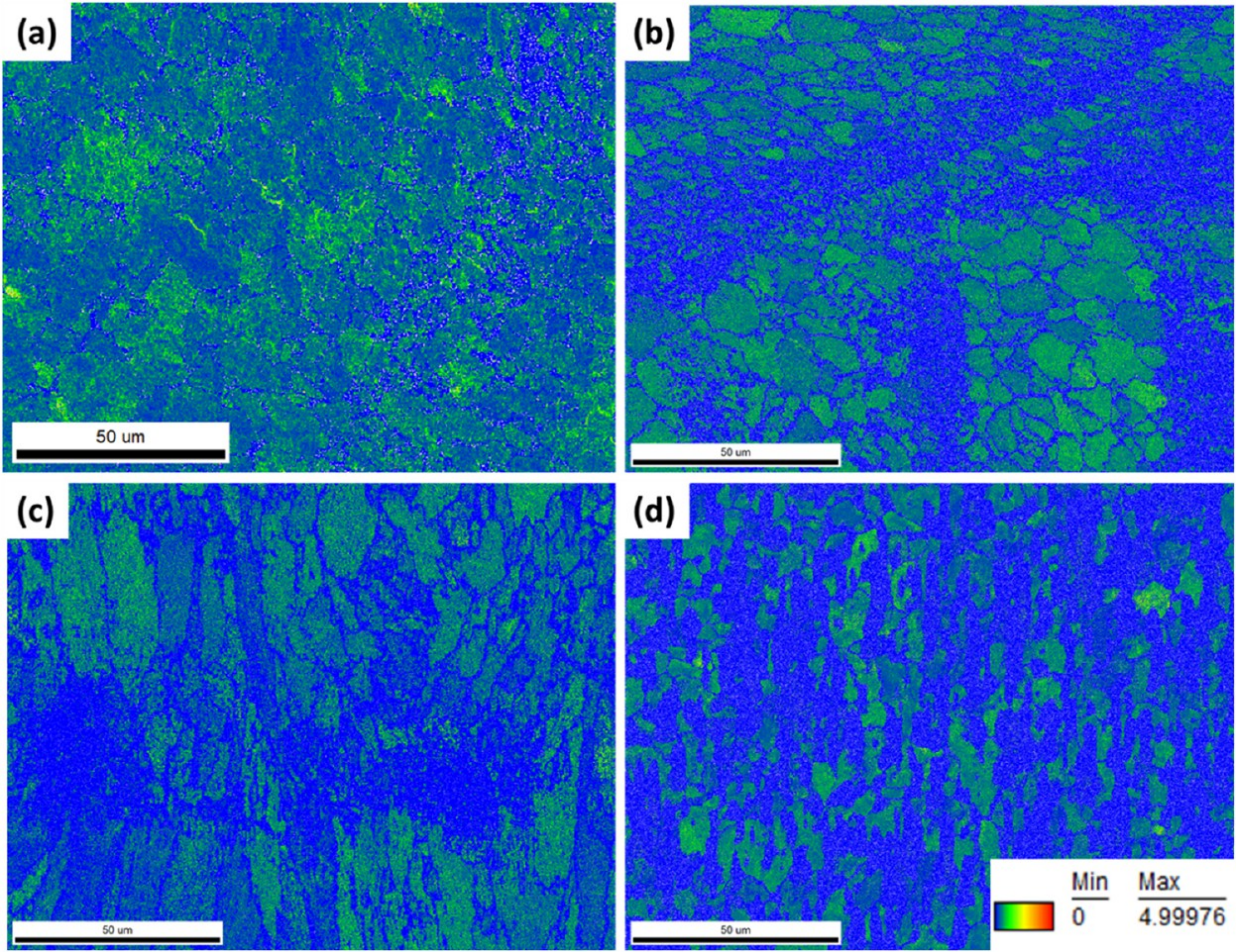
Kernel average misorientation (KAM) maps of LPBF AlSi10Mg in the (a) as-built, (b) T5, (c) SR, and (d) T6 conditions. The color scale represents local misorientation (°), where blue indicates lower stored strain and dislocation density, while green to red regions indicate progressively higher local strain.

In the T5 condition (Figure 6b), KAM values are significantly reduced, indicating effective dislocation annihilation during aging. This relaxation of internal stresses is consistent with the improved ductility and toughness of the T5 alloy, enabling better absorption of the impact energy during erosive wear. The SR condition also demonstrates a reduction in misorientation compared with AB, but localized regions of strain remain along elongated grains. Finally, the T6 sample exhibits the lowest overall KAM intensity, reflecting efficient stress relief and homogenization during two-step treatment. However, this condition lacks the beneficial fine substructures observed in T5.

### 3.5 Texture Development

Figure 7 shows the IPF maps for the different conditions. Each map represents the crystallographic orientation of grains relative to the sample reference axes ([001], [010], and [100]). The color coding corresponds to specific crystallographic directions according to the standard IPF color key, while the numerical scale indicates the relative density of grains aligned along each direction. Higher values correspond to a greater fraction of grains oriented in the crystallographic direction. The comparison among maps (Figure 7a–d) highlights variations in texture and preferred grain alignment across the different processing or heat-treated conditions. The AB alloy shows a relatively weak but discernible texture along the build direction, typical of LPBF-fabricated materials, where epitaxial grain growth occurs due to steep temperature gradients. The texture intensity decreases after heat treatment, with the T5 and SR conditions displaying more randomized orientations. This reduction in texture sharpness contributes to isotropy in the mechanical response, which is advantageous for erosion resistance under varying impact angles. The T6 condition shows moderate texture intensity, particularly along the ⟨100⟩ directions, suggesting preferential recrystallization. While this reduces residual stress, the development of coarser grains aligned with specific orientations may facilitate cleavage-like fracture during impact erosion, contributing to its inferior wear resistance compared to that of T5.

**Figure 7:**
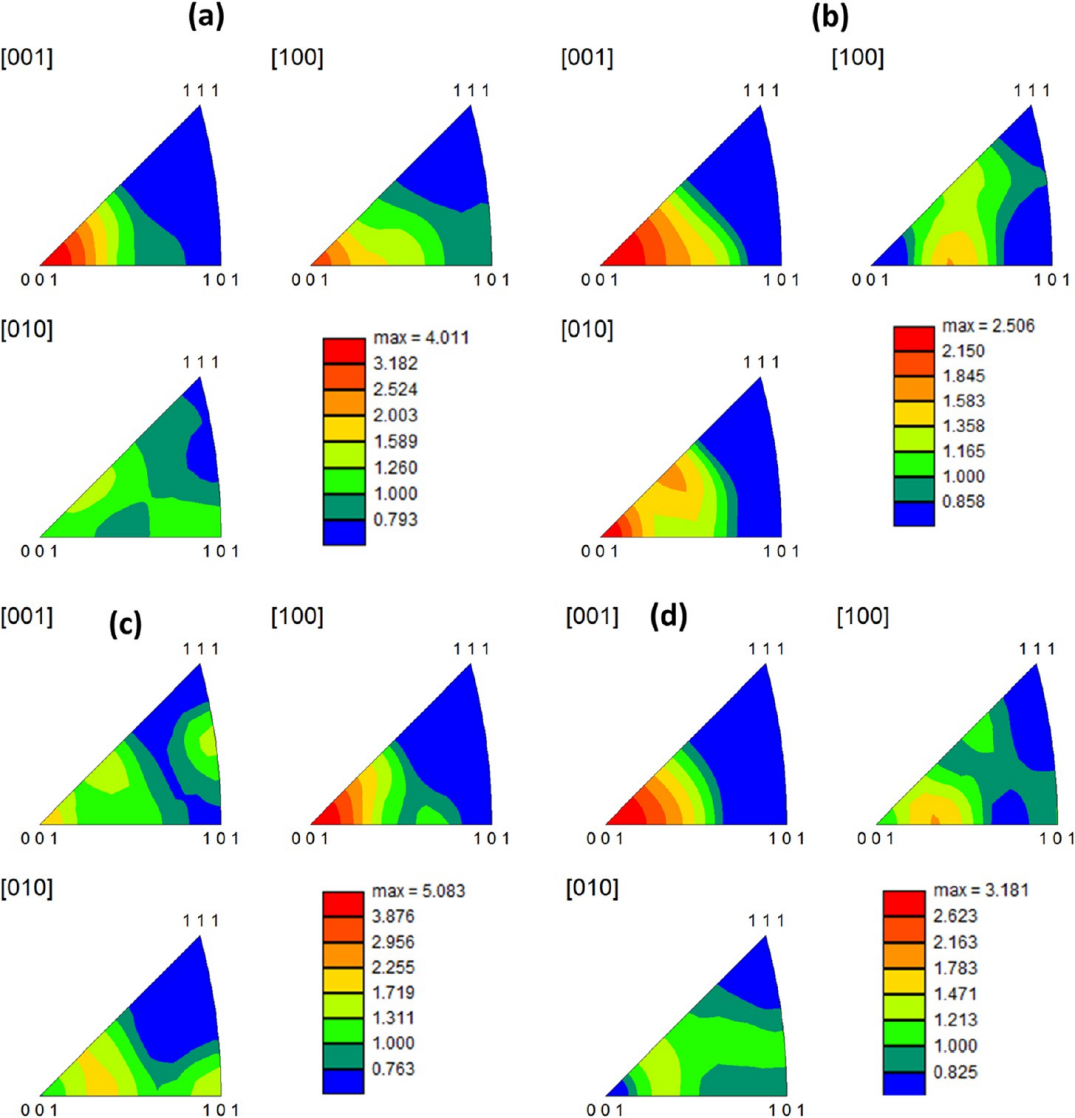
Crystallographic texture maps of LPBF AlSi10Mg in the (a) as-built, (b) T5, (c) SR, and (d) T6 conditions. The color scale indicates the relative orientation density of grains with respect to the sample reference directions.

### 3.6 Microhardness

Figure 8 shows the hardness measurement of different heat-treated samples, where the as-built samples show the highest hardness of ∼136 HV. Following the T5 heat treatment, there is a notable 62.5% reduction in microhardness compared to the as-built sample, resulting in an average hardness value of 85 HV. Similarly, the T6 and SR heat-treated samples exhibit 58 and 48% reductions, respectively, compared to the as-built sample. The as-built samples exhibited increased hardness, attributed to Si particles and a refined α-Al cellular structure, resulting from the rapid cooling manufacturing process.^1^ This fine Al–Si cellular network effectively impedes dislocation motion, providing strong microstructural strengthening. In addition, the rapid cooling rates promote high dislocation density and nonequilibrium solute trapping, further contributing to the elevated hardness.^26,30^ After T5 heat treatment, a large fraction of the fine cellular Si network is retained, while Mg_2_Si precipitates form during artificial aging. The coexistence of the preserved Si network and precipitation strengthening explains why the T5 condition shows the second-highest hardness among all tested states. In contrast, the T6 heat treatment involves solution treatment, which dissolves the original fine cellular microstructure and promotes fragmentation and spheroidization of Si particles. Although Mg_2_Si precipitates form during the subsequent aging step, the breakdown of the continuous Si network reduces the dominant strengthening contribution associated with the as-built microstructure. As a result, the overall hardness of the T6-treated samples remains lower than that of the as-built condition. For the SR heat-treated samples, the primary microstructural change is the onset of Si particle coarsening accompanied by a reduction in dislocation density and residual stress.^31^ Unlike T5 and T6 conditions, no significant precipitation strengthening occurs during stress relief. Consequently, the combined effects of microstructural coarsening and defect annihilation lead to the lowest hardness among all conditions. In general, heat treatment facilitates the relaxation of residual stresses and a decrease in defect density, ultimately reducing the cold work on the samples. Therefore, the observed hardness reduction across all heat-treated conditions can be attributed to the progressive loss of the fine cellular Si network, coarsening of Si particles, and reduction in dislocation density, with the relative contribution of precipitation strengthening depending on the specific heat-treatment route.

**Figure 8:**
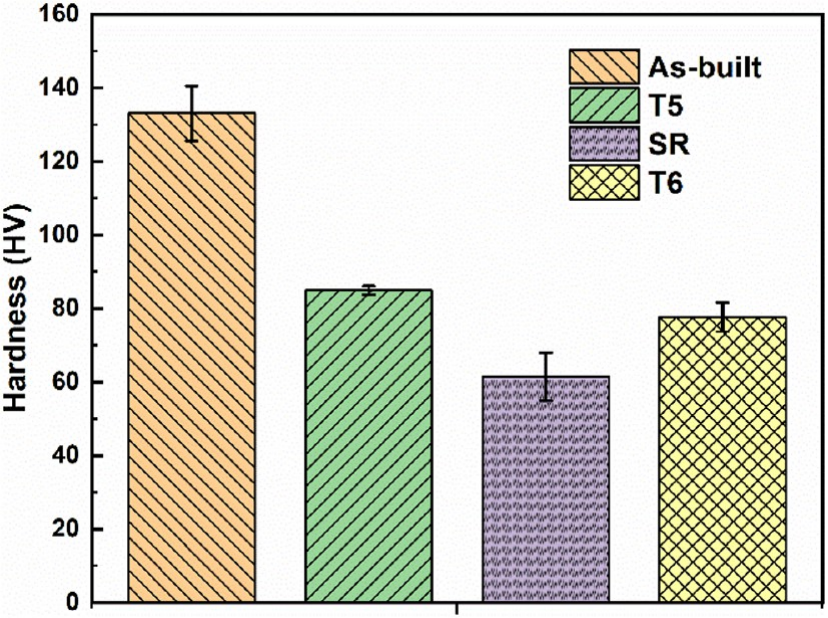
Vickers microhardness of LPBF AlSi10Mg in the as-built, T5, SR, and T6 conditions.

### 3.7 Erosion Behavior

The erosion behavior of as-built and heat-treated L-PBF AlSi10Mg (T5, T6, and SR) samples was evaluated under varying impingement angles of 30, 60, and 90° at a constant particle velocity of 70 m/s. In another set of experiments, the impingement angle was held constant at 30°, while the particle velocity was varied to 30, 70, and 90 m/s to study its effect on erosion response. In addition, the eroded samples were examined using SEM and 3D profilometry to analyze the erosion mechanisms and quantify the volume loss due to erosion.

#### 3.7.1 Erosion Study at Various Impingement Angles

Figure 9 shows the erosive wear rate of the alloy (as-built, T5, SR, and T6) under different impingement conditions (30, 60, and 90°) at 70 m/sec. The erosion rate is severe at 30° compared to 60 and 90°, with the lowest rate observed at 90° for both as-built and heat-treated samples. Stachowiak^32^ reported that if the material exhibits maximum erosion rate at a lower impact angle, it indicates a dominant ductile mode of erosive wear. Conversely, the maximum erosion rate at a higher impact angle (90°) indicates that a brittle mode of erosive wear is dominant. In this research study, the maximum erosive wear observed at 30° suggests that the ductile mode of erosive wear prevails. SEM analysis was carried out to confirm ductile erosive wear on the 30° worn-out samples.

**Figure 9:**
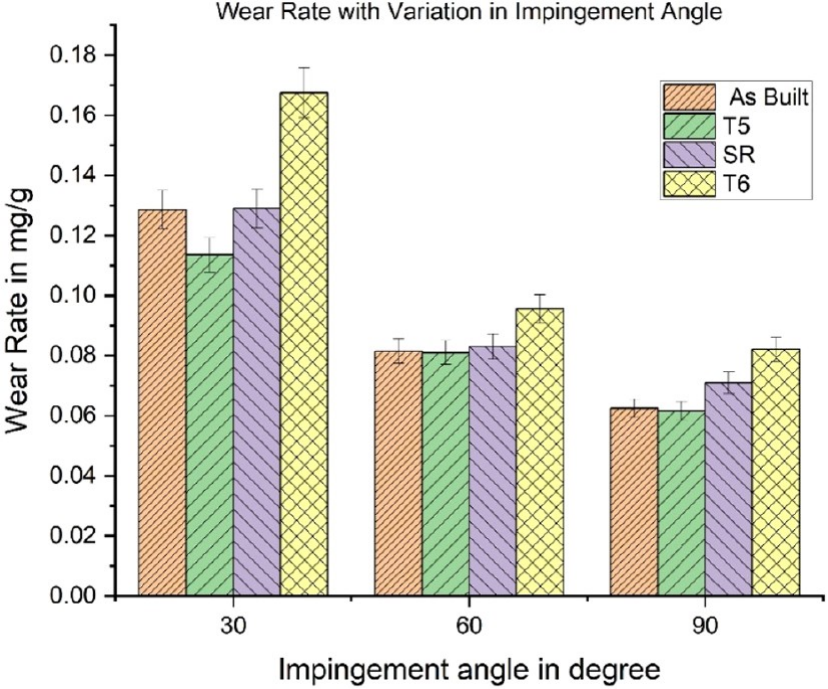
Erosion rate of LPBF AlSi10Mg in the as-built, T5, SR, and T6 conditions at impingement angles of 30, 60, and 90° under a constant particle velocity of 70 m/s. Error bars represent the standard deviation.

The topography analysis shows chipping-out and plowing marks caused by repeated impact of erodent striking a surface. Chipping out (peeled off) in solid-particle erosion typically occurs when the material undergoes plastic deformation. In this phenomenon, the erodent plunges at an angle through the surface, which deforms and peels off material in layers. Later, the peeled-off layer is eventually fragmented under the subsequent impact of the erodent. Also, at a low impact angle, the particle tends to slide across the surface after impact, which results in a wear mechanism similar to abrasion results in plowing. The topography analysis (Figure 10) confirms that the plastic deformation is the predominant mode of material removal at 30° impact angle; however, localized brittle fracture also contributes in the as-built condition, where residual stress and microstructural heterogeneity promote microcrack initiation and fragmentation.

**Figure 10:**
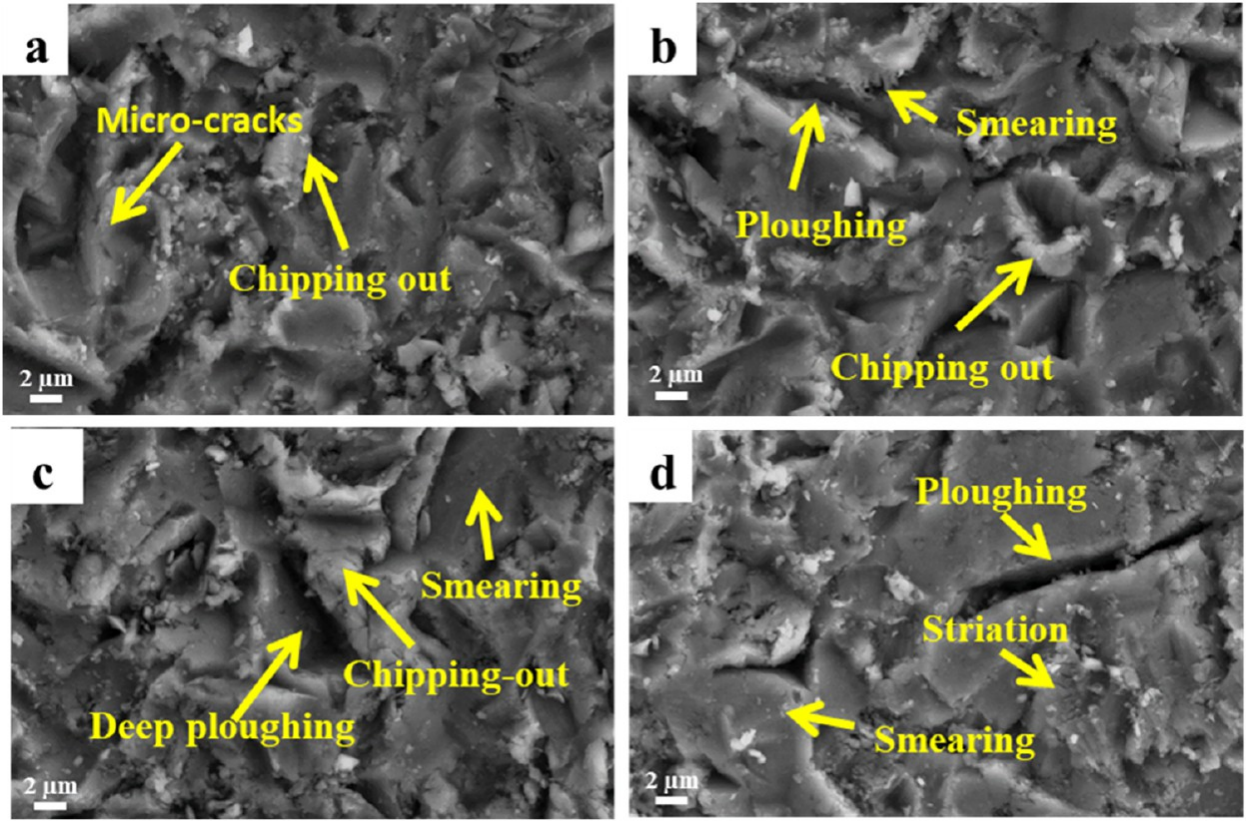
Topography of eroded sample at 30° impact angle: (a) as-built, (b) T5, (c) SR, and (d) T6.

The T5 heat-treated samples exhibit lower erosion rates compared to as-built samples due to improved microstructural stability. While higher hardness in as-built samples can resist the plastic deformation, the rapid solidification process introduces dislocations and residual stresses that make the material prone to flaking and fragmentation under erosive conditions. The microcrack formation in the as-built samples confirms that it is prone to fragmentation. This shows that the residual stress and microstructural stability are also key factors in erosion behavior alongside hardness.^5,33^ Also, the T5 sample exhibits a smearing phenomenon; that is, instead of removing material entirely from the surface, the particle causes the material to spread or smear. The EBSD-KAM analysis further supports this interpretation, where the as-built condition exhibited higher local misorientation and stored strain despite possessing the highest hardness. Under repeated particle impacts, these localized strain concentrations promote crack initiation and fragmentation, leading to increased material removal. In contrast, the T5 condition exhibited reduced local strain accumulation and improved deformation accommodation capability, enabling the material to absorb impact energy through plastic deformation rather than brittle detachment. Therefore, the erosion resistance in LPBF AlSi10Mg is governed by the combined effect of hardness, residual stress state, and microstructural stability.

The erosion rate is severe in the SR and T6 samples due to severe surface damage, which is evident from the topography analysis. The deep plowings, smearing, and striation marks indicate severe plastic deformation involved in 30° impact conditions. However, the SR heat-treated sample shows a significant reduction in erosion rate as compared to T6 samples. It indicates that the stress-relieving controls the fragmentations of chipped-out material and thereby the erosion rate. Overall, the study indicates that at 30° impact condition, the wear loss is predominantly due to shearing actions of the erodent, and the surface damage caused by severe plastic deformation contributes to the wear loss (Figure 11).

**Figure 11:**
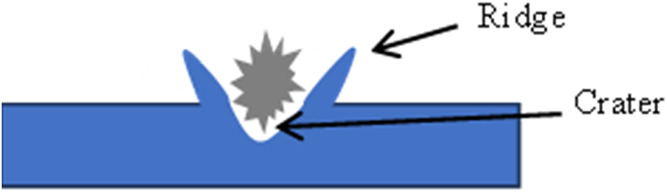
Schematic showing localized plastic deformation under a 90° impact angle.

Under 60° impact angle, the erosion trend (Figure 9), showing similar erosion rates for the as-built, T5, and SR samples, despite differences in hardness, indicates that erosion resistance is influenced by more than just hardness. Microstructural changes caused by heat treatment, such as variations in toughness, fracture behavior, precipitate distribution, and residual stresses, play a significant role in how a material resists erosion. These factors affect how the material absorbs and dissipates impact energy from solid particles, influencing its susceptibility to wear and fracture. Therefore, erosion resistance results from a complex interplay of hardness, microstructure, toughness, and fracture characteristics, not hardness alone. The micrograph (Figure 12) showing slight abrasion marks, blunted ridges, and debris indicates a mixed wear mechanism at this angle. The impact leads to plastic deformation around the crater with the formation of a rim or lip that later becomes flattened due to repeated impacts. At a 60° impact angle, the resultant force of the erodent (solid particle) impacting the surface can be understood as a combination of normal and tangential force components. This results in the observed microstructural damage features like abrasion marks, blunted ridges, and debris. This mixed-mode erosion differs from the sharp cutting at low angles or pure indentation at normal angles, creating a combination of microindentations and plowing wear on the surface.

**Figure 12:**
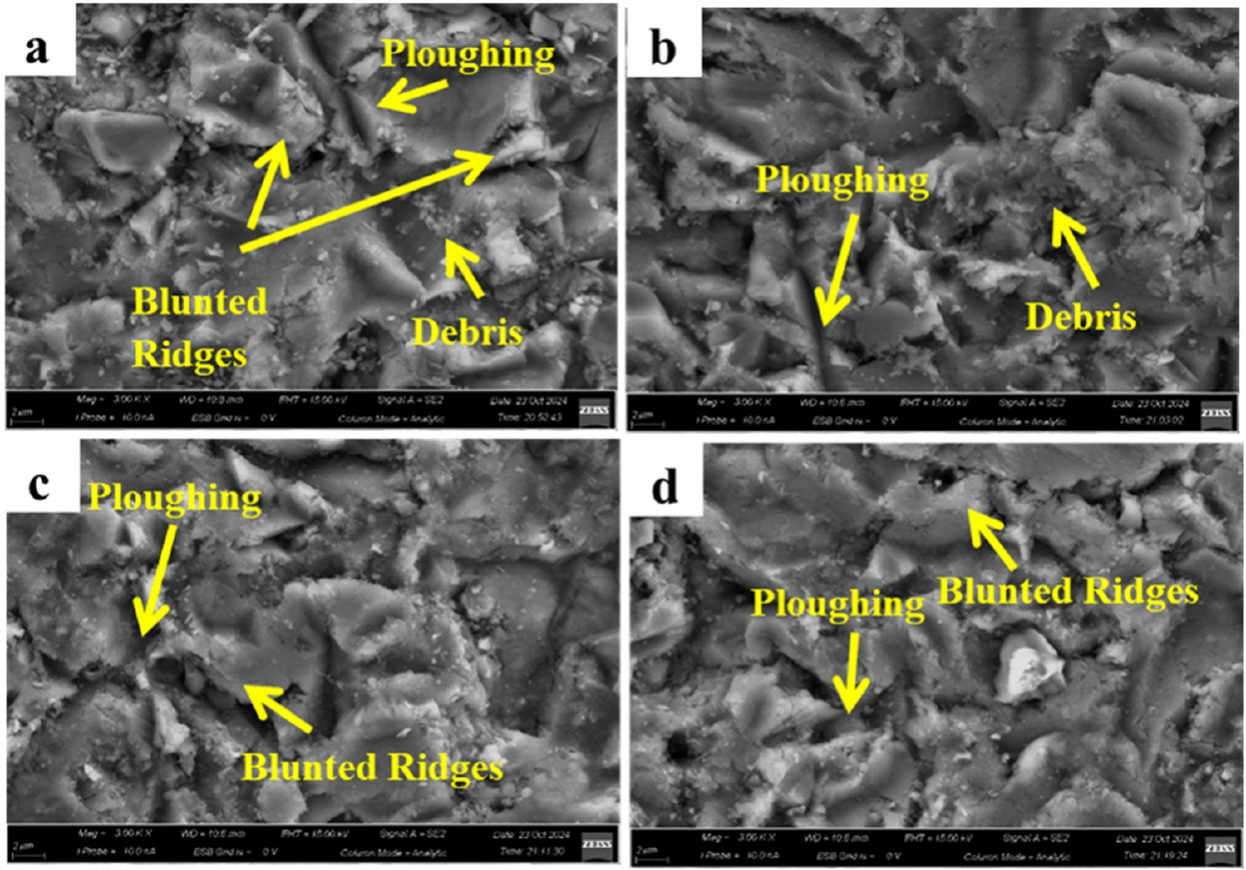
Topography of eroded sample at 60° impact angle: (a) as-built, (b) T5, (c) SR, and (d) T6.

Wear rate graph (Figure 9) shows that at 90° impingement angle, the erosion rate is fairly consistent and lower than at 30°, indicating that the target surface is ductile. As a result, it absorbs the impact of the erodent through localized plastic deformation and forms a crater and ridge, as shown in a schematic representation (Figure 11). The topography analysis confirms ridge formation around the deep crater due to localized plastic deformation in the 90° eroded samples. The erosive wear graph illustrates that the as-built and T5 have the lowest wear rates, while T6 still shows slightly higher wear. The as-built specimen shows debris formed due to the fragmentation of ridges, indicating that the as-built samples are prone to cracking and fragmentation. The T5 sample exhibits the lowest wear rate, with its topography revealing deep craters and ridge formations as evidence of localized plastic deformation. This indicates the T5 sample’s ability to absorb energy through plastic deformation, thereby reducing material loss even under direct impact.

The topography of the SR and T6 samples reveals severe plastic deformation and deep craters, as shown in Figure 13. However, the SR sample distinctly displays plowing marks on the eroded surface, indicating its high ductility, which is proven by the microhardness study. These plowing marks are formed when the eroding particles slide across the surface after impact, causing abrasion and plowing. The T6 samples, on the other hand, showed radial microcrack formation on the ridges. These cracks nucleate at microscopic flaws in highly stressed zones and grow over time, detaching material under consecutive particle impacts.^32^ The formation of microcracks on the ridges resulted in a higher erosion rate compared to other test conditions.

**Figure 13:**
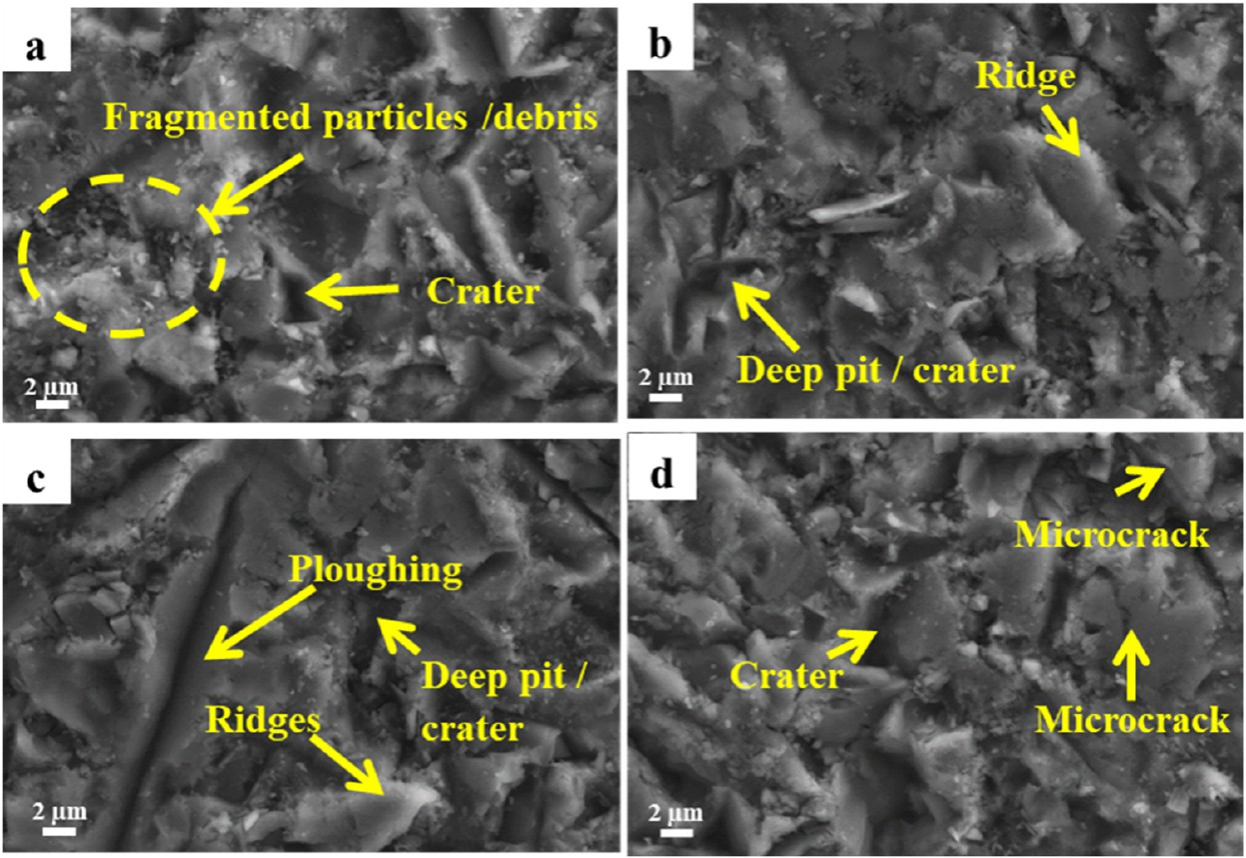
Topography of eroded sample at 90° impact angle: (a) as-built, (b) T5, (c) SR, and (d) T6.

#### 3.7.2 Erosion Study at Various Impact Velocities

The erosion performance of as-built and heat-treated L-PBF AlSi10Mg samples was evaluated for varying impact velocity at a constant impact angle. Figure 14 shows the erosive wear rate of the alloy (as-built, T5, SR, and T6) under different impact velocities (30, 70, and 90 m/s) at an impact angle of 30°. This graph helps analyze how the erosion resistance of each material varies with impact velocity. The wear rate value increases as the velocity increases, reaching its highest value at 90 m/s for as-built and heat-treated samples. With the varying velocity, the T5 samples consistently exhibit a lower wear rate, followed by as-built, SR, and T6.

**Figure 14:**
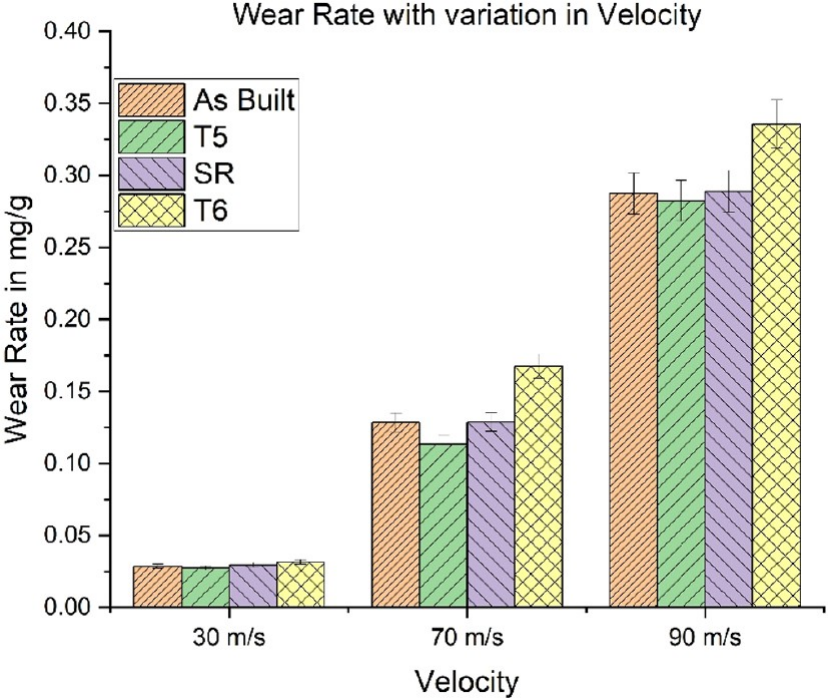
Erosion rate of LPBF AlSi10Mg in the as-built, T5, SR, and T6 conditions at particle velocities of 30, 70, and 90 m/s under a constant impact angle of 30°. Error bars represent the standard deviation from repeated experiments.

The velocity of the erosive particles plays a crucial role in determining the wear process. At low speeds, the impact stress is insufficient to cause plastic deformation and brittle fracture. As the particle speed increases above the threshold, the impacted material may experience plastic deformation. In this regime, commonly observed in many engineering applications, repetitive plastic deformation becomes the dominant wear mechanism.^34^ For brittle materials, wear predominantly results from the subsurface cracking. At extremely high particle velocities, the impacted surface may even undergo a localized melting. Stachowiak^32^ reported that the particle’s impact speed greatly influences the wear rate. Typically, there exists a threshold velocity below which the wear rate is minimal or negligible. For medium to high-speed impact of erodent, the relationship between wear rate and impact velocity can be expressed using a power-law equation^35^

1 $$-dm/dt={\mathrm{Cv}}^{n}$$

where *m* is worn-out material (negative sign indicates material loss), *t* = testing duration (s), *C* = constant, *v* = impact velocity of erodent (m/s), and *n* = exponent velocity (for a solid particle, the “*n*” value ranges between 2 and 3).

Under these testing conditions, as the impact velocity increases from 30 to 90 m/s, the wear rate increases for all materials. This is expected as higher velocities impart more kinetic energy, leading to more severe erosion. The weight loss of test samples under all conditions is relatively low at 30 m/s, indicating minimal erosion at this low velocity. This suggests that at low velocities, the impact energy is insufficient to cause significant damage, resulting in shallow grooves in all test conditions, which is evident from the topography analysis shown in Figure 15. The topography analysis of the samples tested at 70 m/s shows grooves and peeled-off surface as shown in Figure 10. The as-built sample, despite exhibiting higher hardness, shows higher erosion rates compared with the T5 heat-treated samples. The presence of microcracks in the as-built samples indicates a tendency toward fragmentation, reducing their overall erosion resistance compared with the T5-treated samples. The T6 heat-treated sample exhibits the highest wear rate at this velocity, which can be attributed to the microstructural changes induced by the T6 treatment. Despite the relatively higher hardness of the T6 sample, it exhibits higher wear rates compared to the SR samples under the tested erosion conditions. This is due to an increased brittleness and reduced toughness. The microstructural changes in T6 can cause localized stress concentrations, promoting fragmentation during erosion, which is evident from Figure 10d.

**Figure 15:**
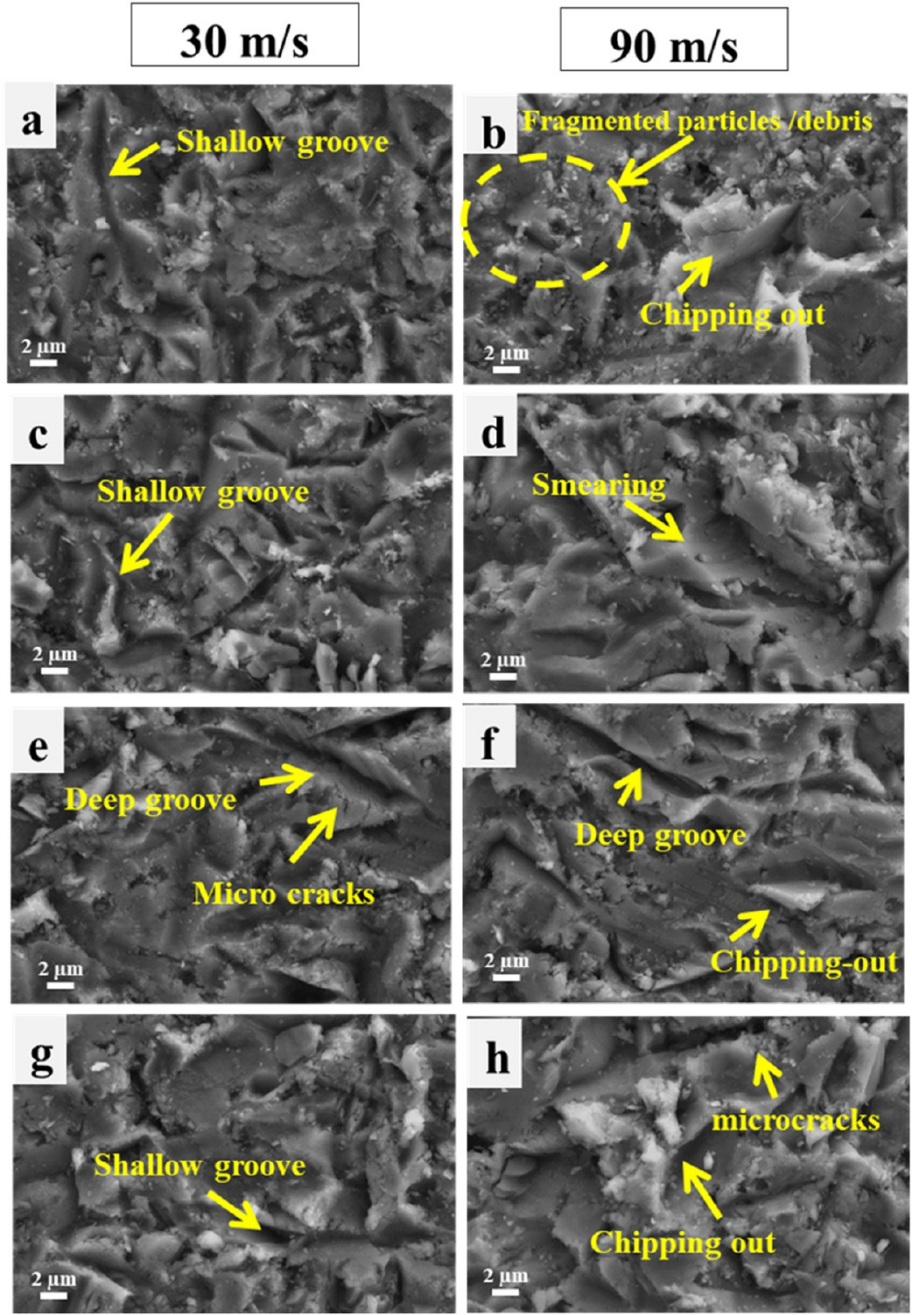
Topography of eroded sample at different impact velocities: (a, b) as-built; (c, d) T5 heat-treated; (e, f) SR heat-treated; (g, h) T6 heat-treated.

At 90 m/s, severe plastic deformation leads to the chipping and smearing of the surface. T6 samples exhibit the highest erosion rate, followed by the as-built conditions, highlighting their relative susceptibility at higher impact velocities. SEM analysis (Figure 15b) confirms brittle fracturing, showing fragmented particles and debris on the eroded surfaces.

The T5 (direct aging) continues to have the lowest erosive wear rate, though the difference between T5 and SR becomes smaller at this high velocity. The SEM analysis (Figure 15d) shows deep groove formation and smearing of surface material, indicating effective absorption of the erodent’s impact energy.

### 3.8 Mechanism

The erosion resistance of the tested L-PBF AlSi10Mg samples exhibited a clear dependence on both the particle impact velocity (30, 70, 90 m/s) and the postprocessing condition (as-built, T5, T6, SR). Across all conditions, the wear (material loss) increased markedly with increasing impact velocity. This trend aligns with general findings in solid-particle erosion research, where the erosion rate (ER) is strongly influenced by particle kinetic energy and often increases in a nonlinear, power-law manner with velocity.^36–38^ Quantitatively, increasing the velocity from 30 to 90 m/s led to more than a 10-fold increase in material removal, highlighting the dominant role of particle kinetic energy in driving surface deformation and material detachment. Among the four microstructural conditions, the T6-treated samples consistently exhibited the highest erosion rates at all velocities. We attribute this to the T6 microstructure; while T6 aging typically increases hardness, it can also reduce ductility and fracture toughness in aluminum alloys. Such embrittlement or reduced toughness makes the surface more vulnerable to crack initiation and propagation under repetitive particle impact. This interpretation finds support in recent studies on particle erosion behavior of Al alloys and composites: for example, in a powder metallurgy study on Al-SiC and Al-WC composites, the authors reported that impact velocity is the dominant factor controlling erosion, with reinforcement content (hence hardness/microstructure) playing a secondary, though significant role.^38^ Their microstructural states preserve a balance of hardness, ductility, and toughness, allowing plastic deformation to absorb impact energy and delay the onset of the surface fracture. This illustrates that hardness alone is insufficient to predict erosion behavior; the interplay of multiple mechanical properties and microstructural features governs resistance to particle impingement.^39^

A quantitative relationship between hardness and erosion rate was evaluated across all processing conditions to support this observation. The analysis shows that erosion rate increases with particle velocity for all samples, with maximum material loss observed at 90 m/s, while the highest erosion consistently occurs at 30° impact angle, confirming ductile-dominated erosion behavior. Although an inverse tendency between hardness and erosion rate is observed, the correlation is not strictly proportional. The as-built condition, despite having the highest hardness (∼133 HV), does not exhibit the lowest erosion rate. In contrast, the T5 condition shows improved erosion resistance even at lower hardness, indicating that microstructural stability and deformation accommodation are more influential than hardness alone. The T6 condition exhibits higher erosion loss despite moderate hardness, which is attributed to microstructural coarsening and increased crack susceptibility under repeated particle impacts. These results confirm that erosion resistance is governed by a combined effect of hardness, microstructure, and impact conditions rather than hardness alone. To further substantiate this trend, a combined hardness-erosion correlation is presented in Figure 16. The plotted data confirm that erosion rate does not follow a strict inverse dependence on hardness, and deviations are evident, particularly for the T6 condition, indicating the governing role of microstructural stability and impact energy over hardness alone.

**Figure 16:**
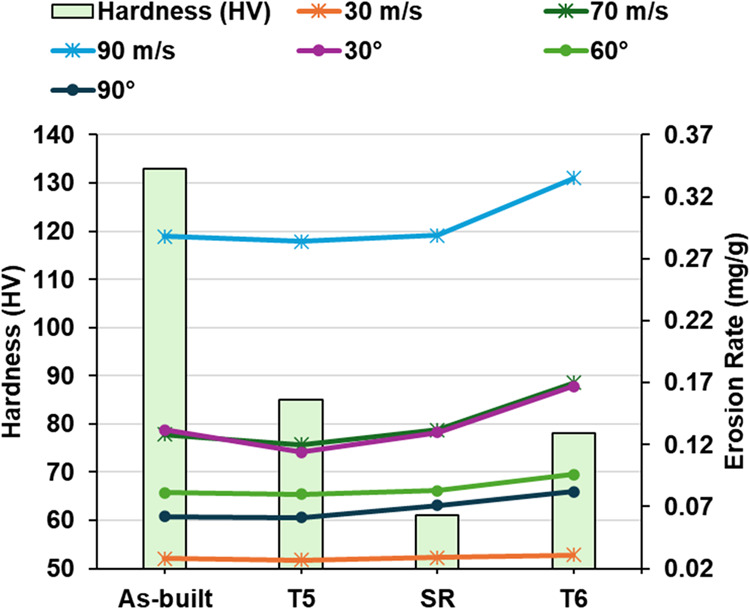
Correlation between Vickers hardness and erosion rate of LPBF AlSi10Mg under different heat-treatment conditions. The comparison includes erosion rates measured at different particle velocities (30, 70, and 90 m/s) and impingement angles (30, 60, and 90°), illustrating that erosion resistance is influenced by both hardness and microstructural stability

This inverse relationship is consistent with empirical observations reported in erosion literature, where the erosion rate (ER) typically follows a power-law relationship with hardness:^39,40^

2 $$\mathrm{ER}=\frac{1}{H^{n}}$$

Here, *H* represents hardness, and the exponent n generally ranges from 1 to 2, depending on the dominant erosion mechanism and material system.

Notably, differences in the erosion behavior between heat-treated conditions were amplified at higher particle velocities. At 30 m/s, wear rates were similar across microstructures, whereas at 90 m/s, the T6 samples showed a pronounced increase in the material removal. This observation emphasizes that the coupling of the microstructural state and impact energy critically influences erosion mechanisms: fine, ductile microstructures can accommodate impact-induced plasticity, whereas coarser or embrittled microstructures fail more readily under high-velocity conditions. Surface morphology analysis supports this mechanistic interpretation. At lower velocities (30 m/s), shallow grooves indicate microcutting-dominated erosion, while at higher velocities (90 m/s), deeper grooves, lip formation, and material delamination reflect a transition to severe deformation and brittle fracture. These observations highlight that the dominant erosion mechanism evolves with particle velocity and is strongly dependent on microstructure and heat treatment conditions. These findings underscore the necessity of selecting postprocessing treatments based on the operational environment. Components exposed to high-velocity particulate flow, such as turbine blades, aerospace ducts, or nozzles, require optimization not only for static hardness but also for dynamic erosion resistance, ensuring that the microstructure can sustain both impact energy and repeated particle-induced damage.

## 4 Conclusions

In this study, the particle erosion wear behavior of AlSi10Mg alloy fabricated via LPBF was systematically investigated. The influence of varying impingement angles (30, 60, and 90°) and particle velocities (30, 70, and 90 m/s) on erosion performance was evaluated. Scanning electron microscopy was employed to examine surface morphology post-erosion, and wear rates were quantified for different sample conditions. Based on the experimental observations and analysis, the following conclusions can be drawn:

- Erosive wear rates in L-PBF AlSi10Mg are highest at shallow impact angles (30°), indicating that shearing-dominated microcutting is most severe at low impingement angles, while higher angles promote deformation- and fracture-controlled mechanisms.
- T5-treated samples consistently exhibit the lowest erosion rates across all impact angles and velocities. This superior performance is attributed to the retention of the fine Al–Si cellular network and the formation of Mg_2_Si precipitates, which together provide an optimal combination of hardness, ductility, and microstructural stability. SEM observations reveal limited groove depth and reduced particle detachment, confirming the microstructure’s ability to absorb impact energy through plastic deformation.
- T6-treated samples demonstrated the highest erosion rates under all test conditions. Despite increased hardness from aging, the solution treatment dissolves the fine cellular network and coarsens Si particles, reducing the fracture toughness and ductility. This makes the surface more susceptible to crack initiation, lip formation, and particle detachment under repeated impacts, as confirmed by SEM topography, showing deep grooves and microcracks.
- The as-built samples, while having the highest hardness, experience significant erosion loss following T6. SEM analysis reveals microcrack formation and localized material removal, highlighting that hardness alone does not govern the erosion resistance. Factors such as residual stress, microstructural stability, and the ability to accommodate plastic deformation critically influence the material response to particle impacts.

Overall, the study demonstrates that the erosion resistance in L-PBF AlSi10Mg is determined by a combination of microstructural features, hardness, ductility, and residual stress. High-velocity impacts amplify differences between heat-treated conditions, emphasizing the need for tailoring postprocessing treatments to achieve an optimal balance of mechanical properties and microstructural stability for service-specific erosion resistance. Furthermore, the present work evaluates the individual influence of impact angle and particle velocity through a sequential experimental approach rather than a fully coupled angle–velocity design, providing fundamental insight into the erosion behavior of LPBF AlSi10Mg under different postprocessing conditions.
